# Internal carotid artery stenosis: A novel surgical model for moyamoya syndrome

**DOI:** 10.1371/journal.pone.0191312

**Published:** 2018-01-11

**Authors:** Jill M. Roberts, Michael E. Maniskas, Justin F. Fraser, Gregory J. Bix

**Affiliations:** 1 Sanders-Brown Center on Aging, University of Kentucky, Lexington, Kentucky, United States of America; 2 Department of Neuroscience, University of Kentucky, Lexington, Kentucky, United States of America; 3 Department of Neurosurgery, University of Kentucky, Lexington, Kentucky, United States of America; 4 Department of Neurology, University of Kentucky, Lexington, Kentucky, United States of America; 5 Department of Radiology, University of Kentucky, Lexington, Kentucky, United States of America; Massachusetts General Hospital/Harvard Medical School, UNITED STATES

## Abstract

Moyamoya is a cerebrovascular disorder characterized by progressive stenosis of the intracranial internal carotid arteries. There are two forms: Disease and Syndrome, with each characterized by the sub-population it affects. Moyamoya syndrome (MMS) is more prominent in adults in their 20’s-40’s, and is often associated with autoimmune diseases. Currently, there are no surgical models for inducing moyamoya syndrome, so our aim was to develop a new animal model to study this relatively unknown cerebrovascular disease. Here, we demonstrate a new surgical technique termed internal carotid artery stenosis (ICAS), to mimic MMS using micro-coils on the proximal ICA. We tested for Moyamoya-like vasculopathies by fluorescently labelling the mouse cerebrovasculature with Di I for visualization and analysis of vessel diameter at the distal ICA and anastomoses on the cortical surface. Results show a significant narrowing of the distal ICA and anterior cerebral artery (ACA) in the Circle of Willis, as observed in humans. There is also a significant decrease in the number of anastomoses between the middle cerebral artery (MCA) and the ACA in the watershed region of the cortex. While further characterization is needed, this ICAS model can be applied to transgenic mice displaying co-morbidities as observed within the Moyamoya syndrome population, allowing a better understanding of the disease and development of novel treatments.

## Introduction

Moyamoya is an occlusive cerebrovascular disorder first reported in 1957 in Japan, and is characterized by stenosis of the supraclinoid portion of the internal carotid arteries (ICA) with the formation of an abnormal vascular network at the base of the brain [1]. Moyamoya is a general term used to describe two different conditions affecting the intracranial internal carotid artery; moyamoya disease (MMD), a congenital disease causing bilateral arteriopathy which is more prominent among East Asian and Japanese children and adults [2], and moyamoya syndrome (MMS), which is idiopathic, and typically seen among Caucasian adults ranging in age from 20 to 40 years. Clinical course for both may be unilateral or bilateral with predisposition to both ischemic and hemorrhagic strokes. While there is no known genetic component in MMS, as there is in MMD, it is often associated with autoimmune disorders such as diabetes, lupus or rheumatoid arthritis [3]. Treatment options for both MMD and MMS involve daily aspirin use, lifestyle modifications to maximize cerebral perfusion, and surgical direct or indirect bypass to restore blood flow.

While MMD has been extensively described, relatively little is known about MMS. Therefore, our goal was to develop a mechanical mouse model that induces Moyamoya-like vasculopathies and to use this model to study the mechanisms of and potential treatments for MMS. For the first time, we show that the application of a micro-coil to the proximal ICA in mice leads to stenosis and hypoperfusion of the distal vessels. This new mechanical internal carotid artery stenosis (ICAS) model could be combined with co-morbid models to help us better understand MMS.

## Materials and methods

### Animals

The experimental protocol was approved by the Institutional Animal Care and Use Committee of the University of Kentucky (protocol #2017–2645) and experiments were performed in accordance with the Guide for the Care and Use of Laboratory Animals of the National Institutes of Health and reported according to the ARRIVE guidelines (S1 File). All analyses were performed in a blinded fashion and animals were randomly assigned (via Research Randomizer online) to treatment groups. All surgeries were performed under ketamine/xylazine anesthesia and all efforts were made to minimize suffering and were performed within the animal facility during the animals light cycle. All mice were housed in a climate-controlled room on a 14/10 hour light/dark cycle (respectively) and food and water were provided *ad libitum*.

This study used C57Bl/6 male mice (16 weeks old) with an N = 11 (n of 6 for the ICAS group and an n of 5 for the control groups). As this was an exploratory study rather than a confirmatory study, power calculations were not used, and smaller groups sizes were favored.

### Surgical preparation and micro-coil placement

#### Preparing surgical materials

Materials used during the procedure include: Suture: three lengths of 4–0 silk suture (Fine Science, 8-S) cut to three inches, one length of 6–0 silk suture (Fine Science, 4-S) cut to one inch, and two lengths of 9–0 nylon suture cut to 0.5 inches (Mani, 2003S); Surgical tools: chest spreader/adjustable wire retractor (Fine Science, 17004–05), one micro-coil (Waken B Technology Company Limited, 540311) with an internal diameter of 0.16 millimeter (mm) and length of 1.5 mm (3 turns), one pair of micro-scissors with a 2 mm cutting edge (Fine Science, 15000–03), one pair of curved forceps (Fine Science, MC40/B), one pair of angled 5/45 forceps (Fine Science, 11251–35) and one pair of straight forceps (Fine Science, 11251–20).

#### Preparing the mouse

**Fig 1:**
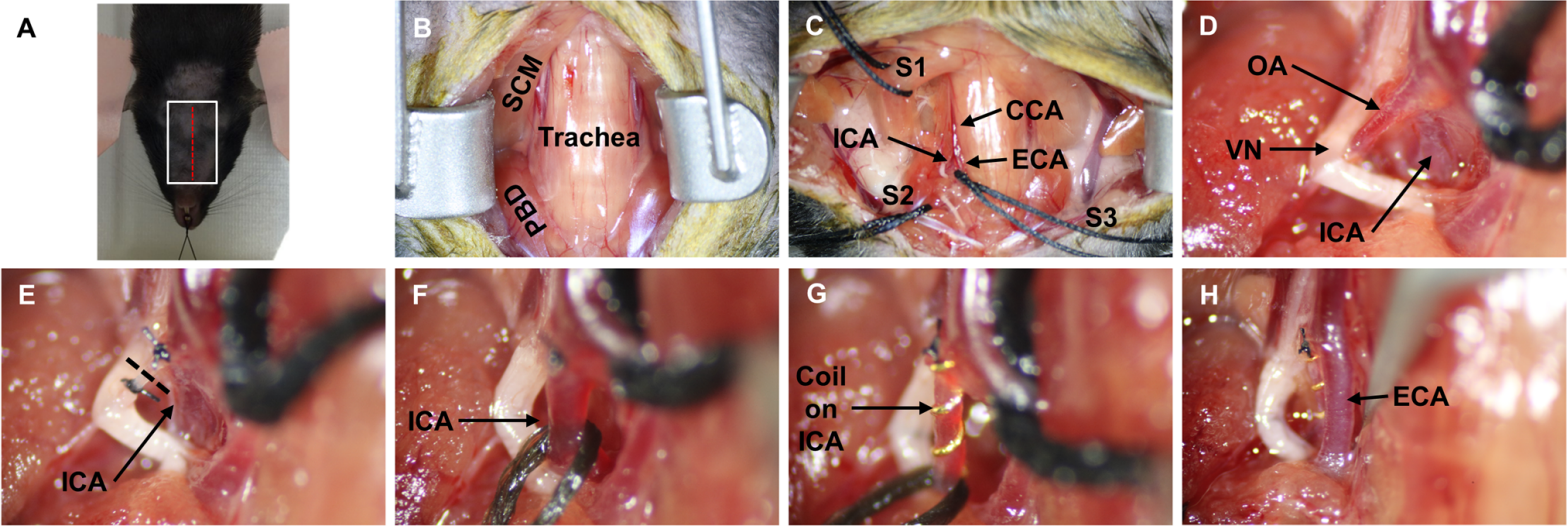
Internal carotid artery stenosis (ICAS) surgical methods. **A)** Orientation of the mouse during the surgical procedure. Head (teeth), forepaws and tail are restrained and incision is made in the midline of the neck (red dashed line). White box indicates region of images that follow. **B)** Opening of the cervical region exposing the trachea, sternocleidomastoid (SCM) muscle and posterior belly of the digastric (PBD) muscle. **C)** Suture (S1-2) placement retracting the SCM and PBD to expose the common, internal and external carotid (CCA, ICA, ECA) arteries. **D)** Identification of the occipital artery (OA), vagus nerve (VN) and ICA. **E)** Suture ligation of the OA and dashed line showing cut to better expose the ICA. **F)** Cut OA with ICA exposed and isolated using 6–0 suture. **G)** Micro-coil placement on ICA deep to ECA (as seen in **H**).

Mice (Jackson Laboratories) were anesthetized with a cocktail of ketamine and xylazine (Henry Schein) using a weight based ratio. Once the mice were anesthetized their cervical region was shaved and the mouse was placed in the supine position with their head, forepaws and tail restrained (Fig 1A). With the mouse in the supine position, the shaved area was cleaned with alcohol and betadine.

#### Opening the cervical region

A midline incision was made from the angle of the mandible to the sternum exposing the trachea, common carotid artery (CCA) and bifurcation of the CCA into the internal carotid and external carotid artery (ICA/ECA). A retractor was used to hold the skin and separated salivary glands from impeding the surgical area (Fig 1B).

#### Retracting the cervical musculature

To increase the visual field, the sternocleidomastoid (SCM) muscle and the posterior belly of the digastric (PBD) muscle were exposed inferiorly and superiorly, respectively (Fig 1B). The tip of a pair of curved forceps was gently placed under the SCM medial to lateral and one length of 4–0 suture was transferred underneath (Fig 1C: Suture 1-S1). The suture was looped around the SCM and secured using tape. This procedure was repeated with the PBD (Fig 1C: Suture 2-S2).

#### Exposing the common carotid, internal carotid and external carotid arteries

With the SCM and PBD temporarily reflected, the CCA was located at its proximal end and gently exposed superiorly to the bifurcation. With the bifurcation identified, the ECA and ICA were exposed superiorly, and the fascia around the vessels was carefully removed. With the ECA exposed and fascia removed around and between the ECA and ICA, the ECA was elevated with curved forceps and one length of 4–0 suture was placed under the ECA (Fig 1C: Suture 3-S3). The suture was looped as performed with the SCM and PBD to lightly retract the ECA medially: the ECA was not occluded but reflected so that the ICA was further exposed.

#### Permanently ligating the occipital artery

With the ICA exposed, the occipital artery running parallel to the ICA was isolated (Fig 1D). As fascia will connect the ICA to the occipital artery, these arteries were next separated by spreading the angled or curved pair of forceps from a closed to semi-open position. With a length of occipital artery exposed, a pair of curved forceps were used to transfer one length of 9–0 suture underneath and place proximally at the ICA origin. The occipital artery was ligated with the 9–0 suture, and this process was repeated at the most distal end of the occipital artery (Fig 1E). Once both sutures were applied, micro-scissors were used to cut the occipital artery fully exposing the ICA (dashed line, Fig 1F). The area around the ICA was cleared of fascia and anything that may have hindered coil placement. For example, a lymph node may be attached to the posterior aspect of the ICA, so curved or angled forceps were used to gently separate the lymph node away from the ICA. With the ICA fully exposed, one length of 6–0 suture was transferred underneath the ICA (Fig 1F).

#### Placing the micro-coil on the internal carotid artery

With the ICA isolated, the 6–0 suture was used as an anchor for coil placement. Fine tipped forceps were used to grasp the coil at one end and place it at an angle to the ICA so that the vessel inserts into the last rung of the coil. With the vessel in the last rung of the coil, the coil was inverted so that it was parallel to the ICA. Using the 6–0 suture, the vessel was gently rotated around the coil so that a length of vessel was placed in each rung of the coil. Vessel placement was assessed to ensure that it was not skipping a rung; if so, the vessel was uncoiled and repositioned until the coil completely encompassed the vessel (Fig 1G).

#### Closing the surgical field and shams

Following coil placement, the 6–0 suture was removed from around the ICA, and the 4–0 sutures were removed from retracting the SCM, PBD and ECA. Saline was applied to the areas previously retracted using cotton tipped applicator. The original midline skin incision was then sutured closed with 4–0 pre-needled silk suture (Ethicon). Sham animals underwent all of the described surgical procedures with the exception of placement of the micro-coil.

### Post-surgical monitoring and euthanasia methods

Immediately following surgery, the mice received an injection (s.c.) of Buprenorphine-SR-Lab (1 mg/ml; ZooPharm) analgesic and were placed in a warm recovery cage. Mice were monitored until fully awake, mobile and interested in food/water and were then placed in their home cage in the animal facility. Mice underwent daily monitoring for signs of sickness or discomfort, including infection at incision site, lethargy, disinterest in food/water, weight loss and seizure. Mice exhibiting these symptoms were immediately euthanized via cervical dislocation/decapitation under ketamine/xylazine anesthetic. For this study, an N = 2 (ICAS group only) died unexpectedly before termination of the study due to unknown causes. At termination of the study (day 28), mice were euthanized by cervical dislocation/decapitation or by perfusion, both under ketamine/xylazine anesthetic.

### Visualization of cerebrovasculature

To visualize the cerebrovasculature, all animals from each group were perfused with the fluorescent dye Di I as previously published [4]. Mice underwent a transcardial perfusion using a perfusion pump (set to 1 ml/min) to perfuse (room temperature) 5 ml of PBS, immediately followed by 10 ml of Di I working solution and then 10 ml of 10% buffered formalin. Brains were carefully removed from the skull ensuring that the Circle of Willis (CoW) remained intact. The extracted brain was then post-fixed overnight at 4°C with 10% buffered formalin. The brains were then transferred into PBS for long-term storage at 4°C, and protected from light.

Fluorescently labelled brains were imaged using a 1 X microscope (Nikon Eclipse E800/Nikon DS-Ril). Images of the cortical vasculature were taken to examine anastomoses and images of the CoW were used to measure vessel diameter. Image analysis was performed using Nikon NES Analysis software to measure vessel diameter (μm). Diameter measurements were taken approximately 20 μm from the bifurcation of the supraclinoid internal carotid artery, M1 segment of the middle cerebral artery, and the A1 segment of the anterior cerebral artery. Anastomoses analysis was performed by counting the number of anastomoses (circle placed over each connection point on a magnified image) between the ACA and the MCA of both the ipsilateral and contralateral hemispheres.

### Statistical analysis

To determine differences between vessel diameters a One-Way Repeated Measures ANOVA was used. To determine significance for cortical anastomoses a *Student’s t-test* was used. Data are presented as the mean ± SEM and significance is indicated by a p-value of * p < 0.05 and ** p < 0.01.

## Results

### Vessel diameter

**Fig 2:**
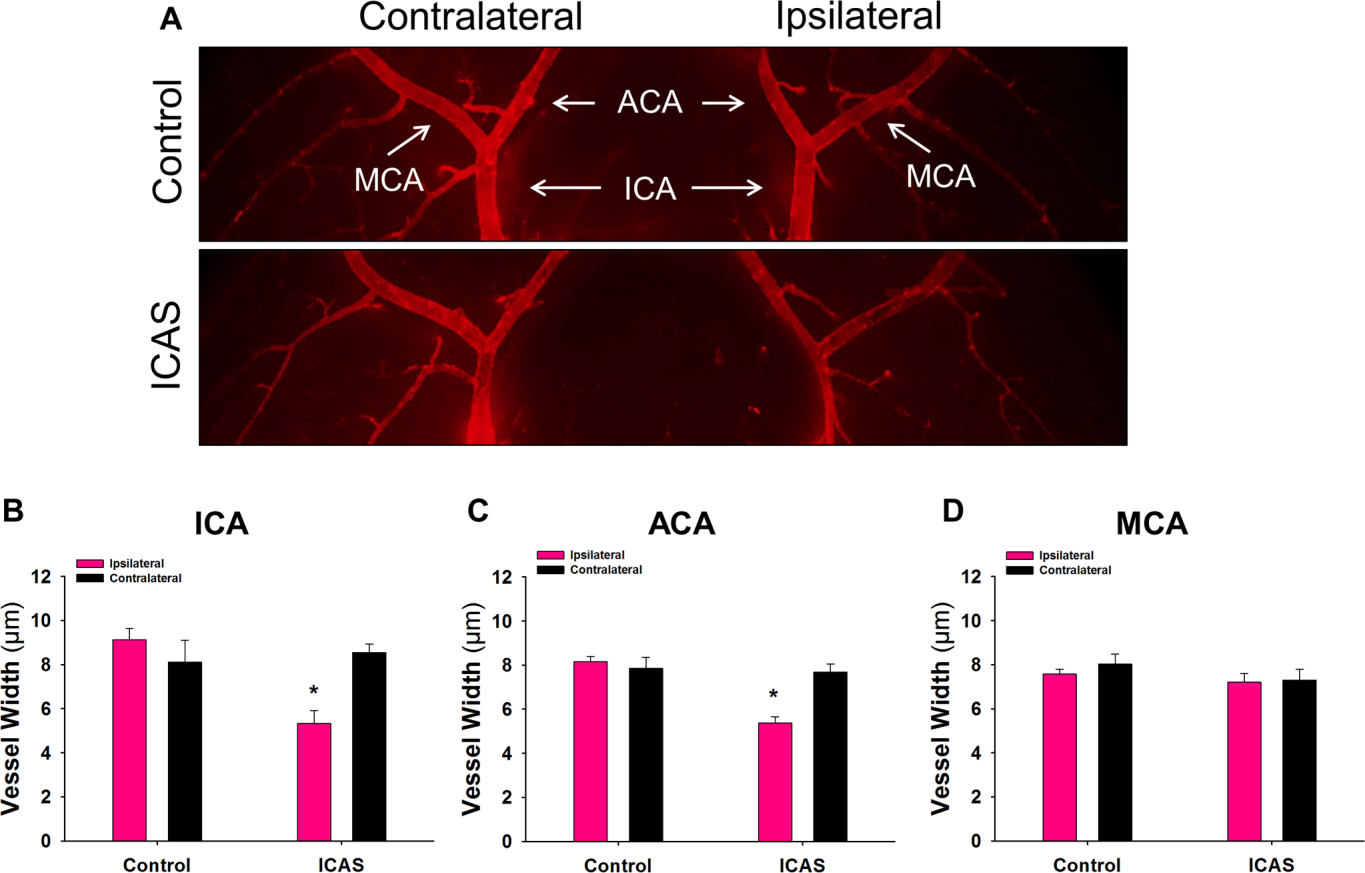
Decreased vessel diameter at the Circle of Willis is observed following ICAS. **A)** Representative images of Di I perfused brains from control and ICAS mice. Images are of the ventral side of the brain and show the ipsilateral and contralateral internal carotid artery (ICA) which bifurcates into the anterior cerebral artery (ACA) and middle cerebral artery (MCA). **B)** Quantification of the ICA, ACA and MCA vessel diameter of the ipsilateral and contralateral sides in control (N = 5) and ICAS (N = 6) mice. *p < 0.05.

Diameters of the ICA, ACA and MCA vessels were examined by measuring the width of each vessel near the bifurcation point on both the ipsilateral and contralateral sides to determine if there was any difference between ICAS and control groups (Fig 2A). Measurements of the distal ICA showed a significant difference between ipsilateral ICAS (5.3 ± 0.6 μm) and ipsilateral control (9.1 ± 0.5 μm) groups (Fig 2B). Similar results were seen in the proximal ACA (Fig 2C), with a significant difference in vessel diameter between ipsilateral ICAS (5.4 ± 0.3 μm) and ipsilateral control (8.2 ± 0.2 μm) groups. Lastly, no difference in vessel diameter of the proximal MCA was noted (Fig 2D).

### Clinical comparison

**Fig 3:**
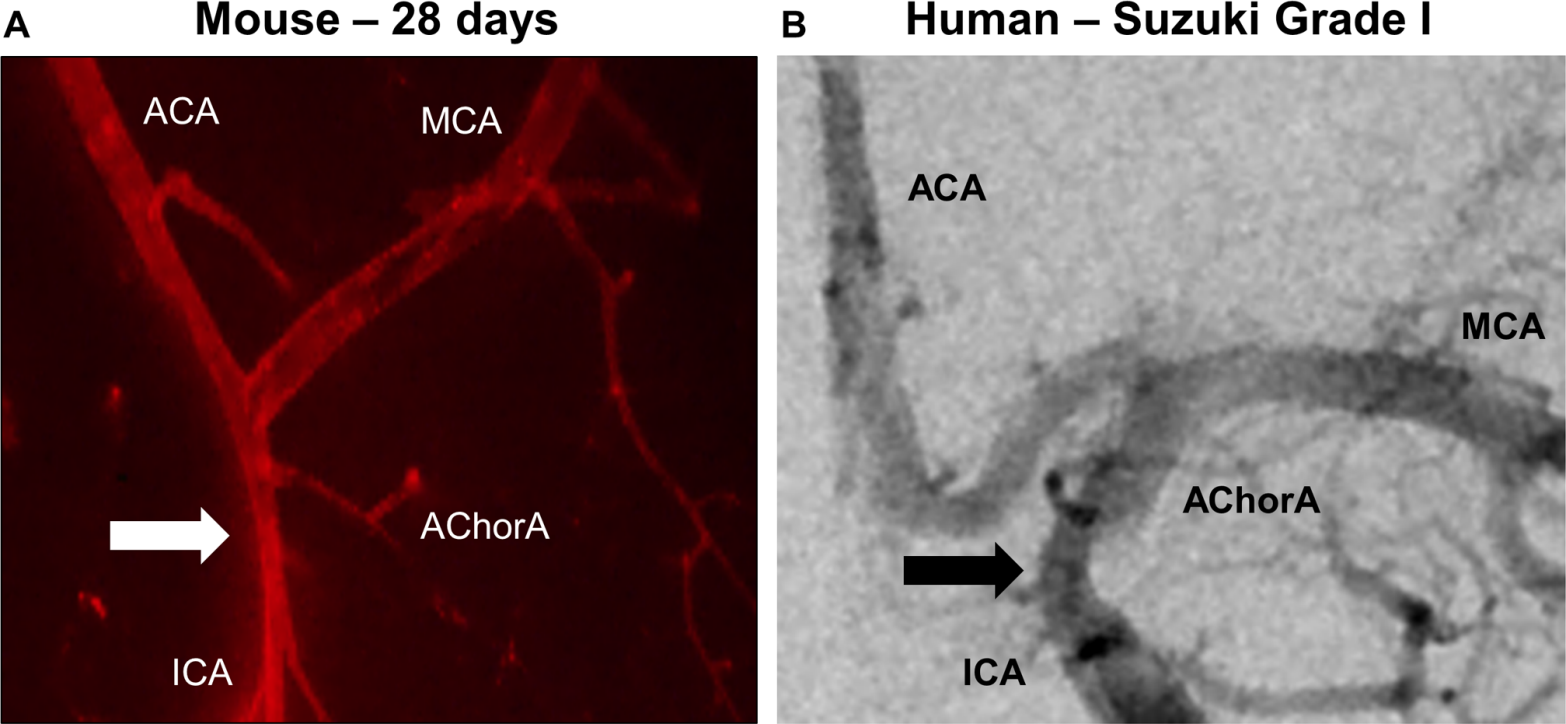
Translational comparison between human MMS and ICAS mechanical mouse model. **A)** Image of Di I stained blood vessels from ICAS mouse model compared to **B)** AP angiogram of de-identified patient with Suzuki grade I MMS. Block arrows indicate the supraclinoid ICA stenosis, similar in position in both images. AChorA: anterior choroidal artery.

To determine whether cerebrovascular changes induced by ICAS might mimic human MMS, we qualitatively compared our ICA, ACA, and MCA vessel diameter measurement results to that seen in clinical MMS. Fig 3 demonstrates a narrowing of the ICA proximal to the bifurcation into the ACA and MCA in both our mouse model of ICAS (Fig 3A) and a patient that presented with a Moyamoya Suzuki Grade 1 (Fig 3B), classified on a scale of 1 to 5 according to degree of stenosis and grade of abnormal neovascularization (see discussion).

### Cortical anastomosis

**Fig 4:**
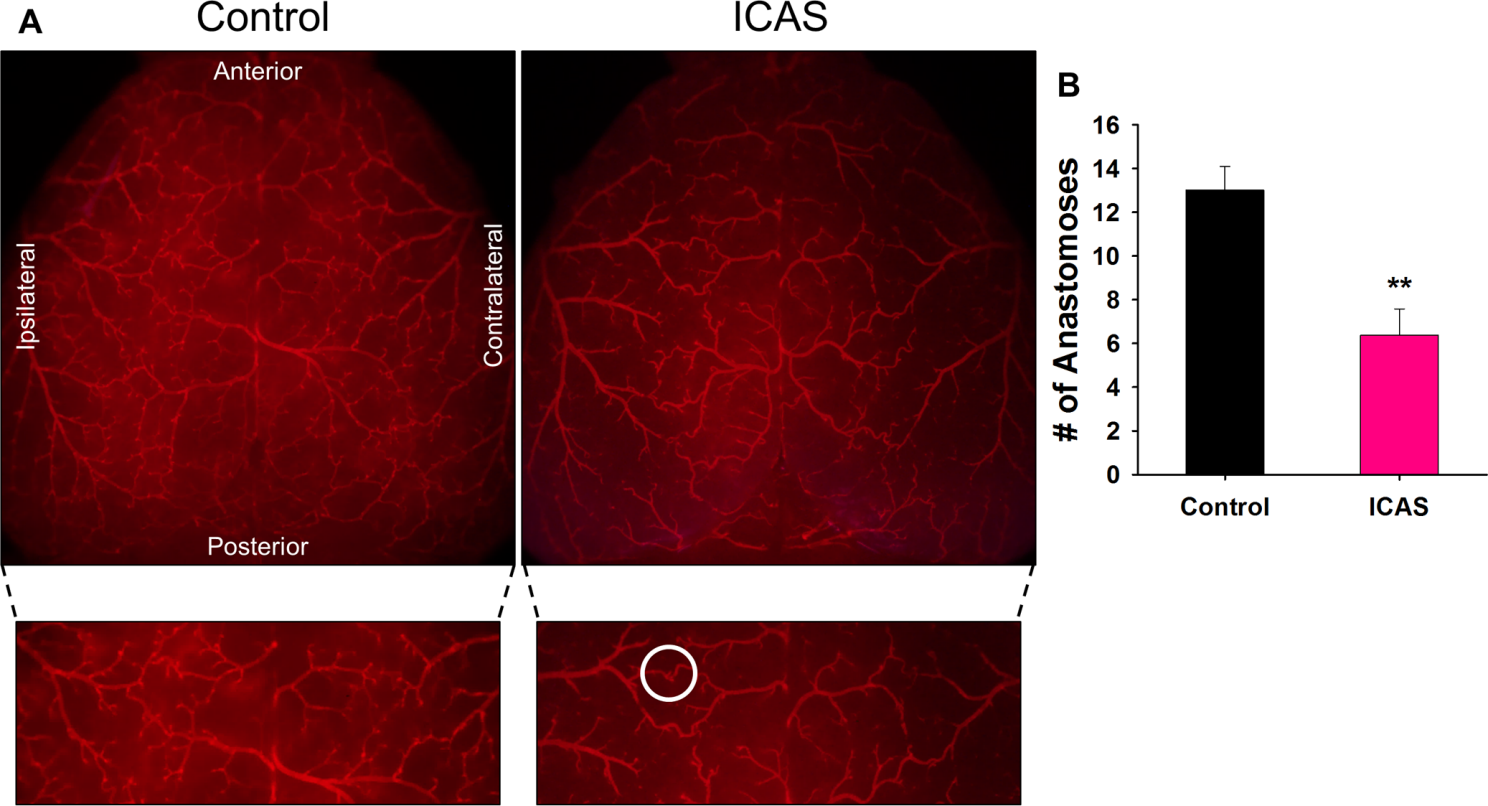
Decreased number of anastomoses in ICAS mice. **A)** Representative dorsal images of Di I perfused brains of control and ICAS mice. Magnified images below to show detail of branching and connections. White circle is one example of an anastomosis. **B)** Quantification of the number of anastomoses between the ACA and MCA of both ipsilateral and contralateral sides in control (N = 5) and ICAS (N = 6) mice. **p < 0.01.

Images of the blood vessels on the cortical surface of the brain were taken following Di I perfusion to examine the number of anastomoses between the MCA and ACA branches (Fig 4A). A significant decrease in the number of anastomoses in both the ipsilateral and contralateral hemispheres was observed in the ICAS (6.3 ± 1.2) group when compared to the control (13.0 ± 1.1) group (Fig 4B).

## Discussion

Moyamoya is a cerebrovascular disorder characterized by progressive stenosis of the intracranial internal carotid arteries leading to both hemorrhagic and ischemic strokes [2, 3, 5]. Restriction of blood flow through the ICA leads to eventual development of new blood vessels resembling a “puff of smoke” (moyamoya in Japanese) in the subcortical region. For years, moyamoya was used as an umbrella term to categorize individuals with stroke-like symptoms presenting with ICA stenosis, but recently it was determined to occur as either a disease or syndrome, the later likely secondary to other underlying causes. Principally affecting women (70–85%) more than men (15–30%), moyamoya spans ethnicities, but is most prevalent in East Asians and Caucasians. Moyamoya disease (MMD) is prominent amongst the East Asian population presenting in both children and adults with a familial lineage [1, 6]. Moyamoya syndrome (MMS) is prominent amongst Caucasians in the 2^nd^/3^rd^ decades of life, is idiopathic, and usually presents with co-morbidities (autoimmune diseases) [2, 7, 8]. Clinical literature often does not distinguish between those with MMD and MMS. Since there is no animal model available to study MMS, unlike the genetic animal model for MMD [9, 10], we set out to create a mechanical model that could ultimately be combined with a co-morbid animal model in order to better understand MMS. Therefore, we developed the ICAS procedure in which a micro-coil was applied to the left ICA of a mouse to induce unilateral hypoperfusion. This led to Moyamoya-like vasculopathies.

The application of micro-coils to induce cerebral hypoperfusion is well documented, most notably in the bilateral common carotid artery stenosis (BCAS) procedure [11, 12]. Specifically, BCAS involves the application of two micro-coils on the common carotid arteries for varying durations to induce a wide range of vascular and cognitive changes. Shibata et al. presented data from a range of coil diameters (0.16–0.22 mm ID) and found that while the 0.16 mm ID coil produced severe pathologies, it had the highest mortality rate, and led them to recommend use of the 0.18 mm ID coil for model reproducibility [12]. Using BCAS as a starting point, we further refined the application site (ICA vs. CCA), the coil diameter (0.16 mm; since the ICA is more narrow than the CCA requiring a smaller diameter coil to become sufficiently stenosed), the coil length (1.5 mm, 3 rungs; due to the physically accessible region in which to apply the coil), and the surgical technique to determine if we could specifically alter the diameter of the distal ICA and proximal ACA, thereby decreasing blood flow unilaterally. Thus far, the mortality rate with the 0.16 mm ID coil on the ICA is less than 10%, though further investigation with various coil diameters is needed. The creation of a mechanical induction of unilateral hypoperfusion to mimic MMS fills a research void in an understudied syndrome.

Current research on moyamoya focuses on MMD, specifically on a genetic mutation (RNF213) affecting the endothelial cells [13–15], which is consistent with the disease seen clinically in the East Asian population. However, we believe a mechanical model is needed to study the pathogenesis of MMS where this genetic mutation has not been implicated. Using a micro-coil placed on the ICA for 28 days, we have mimicked the clinical cerebrovascular condition of early stage MMS in 16 week old male mice (~26 year old person), corresponding to the age range (20–40 years) at which a person might develop MMS. This includes a narrowing of the distal ICA and downstream blood vessels and a decrease in the number of anastomoses within the watershed region of the brain, reflecting Moyamoya-like vasculopathies. A variety of techniques are available to examine blood vessels, such as space-occupying materials (India ink, FITC, latex), magnetic resonance angiography (MRA), and two-photon microscopy. However, they are often technically demanding, require expensive equipment, or the dye molecules may freely move (with pressure from tissue processing) because they are not bound to the blood vessels. Perfusion with Di I, as performed here, directly stains the endothelial cell membranes and thus produces a high signal intensity, and is an efficient, low cost alternative technique for examining the vasculature [4].

Moyamoya is characterized through cerebral angiography, performed to evaluate the severity of the stenoses, along with the extent of collateral networks. Based on angiographic assessment, the Suzuki grading system is used to document the severity of the disease, ranging from Grade I (narrowing of the ICA apex) to Grade V (Occlusion of the ICA and disappearance of moyamoya collateral vessels) [16]. While our evaluation of the model thus far only included a short time point (28 days), it mimicked the findings suggestive of Suzuki Grade I Moyamoya, which is not characterized by lenticulostriate and/or pial neovascularization and collateral formation. Further studies with extended time points and functional outcome measurements are necessary and planned to characterize the model and severity of the condition in order to understand how the model can mirror the progression of the clinical condition.

In addition, there are other limitations to our study. While patients do present with unilateral pathologies, bilateral ICA coil application may accelerate the model, or change the dynamics of pathology development. Given that the syndrome can and often is bilateral, this will be important to perform in future studies. Examination of female mice may also provide further insight into disease progression as females have a higher incidence of MMS as previously mentioned. While Di I is a reliable method to label the vasculature, it is possible that the Di I was not able to penetrate all vessels of the brain (mainly watershed region) due to stenosis/occlusion or differences in perfusion pressure from one vessel to another. Further studies utilizing multiple techniques (such as MRA) to evaluate the cerebral vasculature are needed to further characterize this model. Additionally, it will be necessary to evaluate the intraluminal changes in the cervical and intracranial carotid artery at long intervals in order to understand the inflammatory changes of the model compared to known features in clinical moyamoya. Furthermore, MMS is idiopathic but predominantly presents with co-morbidities, specifically autoimmune diseases (e.g. Lupus, Rheumatoid Arthritis). To further understand MMS and better mimic the clinical condition, micro-coils should be placed on a transgenic mouse that expresses an autoimmune disease typically associated with MMS. By marrying the two, one can gain a better understanding of disease progression and potential treatment(s). Despite these limitations, our study clearly delineates a new animal model that shows early promise as a research tool in a disease for which there are no current standard animal models.

In conclusion, animal models are critical to improving our limited understanding of MMS pathology and to develop new treatments. Here we demonstrate for the first time a novel surgical model using micro-coils on the internal carotid artery that induces early pathologic changes similar to the human condition in healthy mice. While more extensive evaluation is needed, this methodology could provide a key tool for studying MMS in the laboratory.

## Supporting information

S1 File The ARRIVE guidelines checklist. (PDF)
